# Mapping the mortality-to-incidence ratios of Alzheimer’s Disease and Related Dementias (ADRDs): Evidence from the South Carolina Alzheimer’s disease registry

**DOI:** 10.1371/journal.pone.0339785

**Published:** 2025-12-29

**Authors:** Eric Mishio Bawa, Daniel A. Amoatika, Margaret C. Miller, Bankole A. Olatosi, Lorie Donelle, Sue E. Levkoff, James W. Hardin, Xiaoming Li, Leonardo Bonilha, Daniela B. Friedman, Swann Arp Adams

**Affiliations:** 1 Department of Epidemiology and Biostatistics, Arnold School of Public Health, University of South Carolina, Columbia, South Carolina, United States of America; 2 Office for the Study of Aging, University of South Carolina, Columbia, South Carolina, United States of America; 3 Department of Health Services Policy and Management, Arnold School of Public Health, University of South Carolina, Columbia, South Carolina, United States of America; 4 Biobehavioral Health and Nursing Science Department, College of Nursing, University of South Carolina, Columbia, South Carolina, United States of America; 5 College of Social Work, University of South Carolina, Columbia, South Carolina, United States of America; 6 Department of Health Promotion, Education, and Behavior, Arnold School of Public Health, University of South Carolina, Columbia, South Carolina, United States of America; 7 Department of Neurology, School of Medicine, University of South Carolina, Columbia, South Carolina, United States of America; Nathan S Kline Institute, UNITED STATES OF AMERICA

## Abstract

**Introduction:**

Mortality-to-incidence ratios (MIRs) are useful in assessing disease burdens and illustrating disparities. Unlike cancer, MIRs have not been applied to ADRDs. Therefore, we estimated and mapped the MIRs for ADRDs to show disparities in South Carolina.

**Methods:**

Using data from the South Carolina Alzheimer’s Disease Registry (2017−2021), ADRD MIRs were calculated by demographic and geospatial characteristics. To account for the influence of the COVID-19 pandemic, data from 2015 to 2019 were also examined. MIRs were calculated as age-adjusted mortality rates divided by age-adjusted incidence rates.

**Results:**

Overall, Black people and rural individuals consistently experienced higher MIRs, with the COVID-19 pandemic increasing this disparity gap. MIRs greater than 1.00 were only observed among Black people. The MIR for 31 out of 46 counties exceeded the state average.

**Discussion:**

Estimating and mapping ADRDs has aided in identifying specific areas with the greatest burden of ADRD in South Carolina for targeting interventions.

## Introduction

Alzheimer’s disease is the leading form of dementia and a public health priority [1]. In addition, it is one of the top 10 leading causes of death in the United States (US) [1,2] and the fifth-leading cause of death among American older adults [2]. In the US, about 6.9 million people are living with Alzheimer’s disease in 2024 [2] and this number is forecasted to double by 2060 [2]. This is partly due to the aging population of baby boomers, who represent the largest generation in the US and began reaching age 65 in 2011. Among the 6.9 million older Americans with Alzheimer’s disease, about 4.2 million are women [3].

The incidence and prevalence of Alzheimer’s disease both differ by race and ethnicity in the US. Non-Hispanic Black and Hispanic older adults are more likely to develop Alzheimer’s Disease and Related Dementia (ADRD) than non-Hispanic White older adults [4]. It has previously been reported that Black older adults were about two times more likely to develop ADRD compared with their White counterparts [5–7]. Additionally, older Hispanic individuals are approximately 1.5 times more likely to develop ADRD as compared to White individuals [2].

The South Carolina Alzheimer’s Disease Registry (SCADR) is the oldest and most comprehensive of the four statewide Alzheimer’s disease registries. Using data from the registry, researchers can estimate the prevalence of ADRD for planning and healthcare services [8] and the data have been used to inform programs, interventions, and policy [9]. We know that in 2021, 122,699 individuals with ADRD were living in South Carolina, with about 11% of the population aged 65 years and above having ADRD [9]. Orangeburg County in South Carolina was ranked among the top 10 counties in the US with the highest prevalence of Alzheimer’s disease in 2020 [10]. It is the second-largest county in South Carolina by total area, with a population of approximately 84,233, representing about 20% of the state’s older adults aged 65 and above [11].

During the height of the COVID-19 pandemic, Alzheimer’s disease was a significant comorbidity, likely due to pathological changes like the overexpression of pro-inflammatory molecules in patients with Alzheimer’s disease [12]. Researchers using an observational study design reported a higher proportion of COVID-19 cases among the Alzheimer’s disease population [13]. Also reported was an elevated likelihood of COVID-19 infection and mortality among individuals with Alzheimer’s disease [12], with evidence attributing this association to a shorter survival probability from symptom onset compared to the general population [14]. Research analyzing mortality among individuals with ADRD during the COVID-19 pandemic in the US revealed a significant rise in excess deaths from March 2020 to February 2021. Notably, there were substantial racial disparities, with Black adults experiencing the highest levels of mortality [15].

Comprehensive data are limited on ADRD burden by race and region using a population-based indicator, such as the mortality-to-incidence ratio (MIR). The mortality-to-incidence ratio (MIR) provides a metric to assess the efficacy of chronic disease control programs [16,17]. In contrast, traditional mortality statistics are expressed in terms of the underlying total population (usually derived from census reports) which includes individuals with and without the underlying disease. Thus, mortality rates do not account for those diagnosed with the disease and are at risk of dying from it. Consequently, the full burden of disease can be attenuated with traditional mortality rates.

Previous studies used the MIR statistic to identify racial disparities in cancer and evaluate the association between healthcare systems and cancer outcomes [18–20]. Using data from the South Carolina Central Cancer Registry, Hébert, et al reported racial differences in MIR for cancer, with higher MIR observed among Black people compared to White people [18]. A study on national cancer management policies reported a linear relationship between MIR and health system rankings. Lower than predicted MIRs for cancers suggested a stronger national control policy such as cancer screening [19]. Another study on colorectal cancer found an association between the quality of healthcare systems and MIR, suggesting that the MIR could be a useful indicator for both treatment and identifying disparities [21]. The use of the MIR has therefore strengthened public health practice through identification of disparities, evaluating control programs and developing targeted interventions among others.

The incidence and mortality of ADRD vary across regions [22] and may be influenced by factors such as demographics, availability of diagnostics and access to health care. For instance, in rural communities, the incidence of ADRD may be influenced by under-ascertainment of incident cases. Because the MIR provides an avenue to identify regions with higher or lower mortalities, normalized to their incidences [23], extending this metric to ADRDs will aid in the identification of regions in need of timely and focused attention for the allocation of public health resources. In addition, the MIR provides a standard population-based approximation of survival across regions and races without resorting to survival studies, which are time-consuming, expensive, and strained with logistical challenges concerning patient follow-up and minority participation. Estimating the MIRs for ADRD using a population-based registry provides an opportunity to assess the burden of ADRD across the state and individual counties, identifying geographic regions where individuals might experience significantly different outcomes post-ADRD diagnosis. Research examining the MIR of ADRD using data from such a comprehensive registry is sparse. This study estimates MIRs for ADRD by race, region, and county using data from the South Carolina Alzheimer’s Disease Registry.

Despite differences in survival and competing risk, MIR is informative for ADRD because it serves as a valuable proxy for identifying disparities and provides an intuitive understanding of the diagnostic and care processes. Beyond lethality, a higher MIR is also reflective of situations such as systematic or continuous under-diagnosis of new ADRD cases, regional difference in death certification where ADRDs is more frequently listed as the underlying cause of death in some regions than others, and survival differences post diagnosis where certain populations or groups experience higher mortality rates because of late diagnosis or less effective care or comorbidities. MIR was chosen over the mortality-to-prevalence ratio because MIR allows us to simultaneously examine the occurrence of new cases and deaths, making it possible to ascertain the differences in detection patterns and the timing of diagnosis. In addition, the MIR is robust to the duration of disease and extensively used in other studies [18,20,21,23].

## Materials and methods

### Data and analytic sample

This study was a cross-sectional analysis of data from the South Carolina Alzheimer’s Disease Registry, a comprehensive registry of South Carolina residents diagnosed with ADRD. The registry incorporates data from sources such as vital records, long-term care evaluations, emergency department visits, Medicaid claims, memory clinics, and inpatient hospitalizations [24]. This study utilized data from the most recent five-year period available in the registry, 2017–2021 (N = 62,590). To account for the influence of the COVID-19 pandemic, data from the latest five-year period before the COVID-19 pandemic in the USA, 2015–2019 (N = 64,064) were also examined. The study population was restricted to individuals aged 50 years and older. Hispanic populations were not included in this study because the sample size of this population was insufficient (<5%) for reliable stratified analysis. All data were fully anonymized before access by the researchers. Data is anonymized by the South Carolina Revenue and Fiscal Affairs (RFA) and transmitted via a secure FTP network to the Office of the Study of Aging. Therefore, the process of anonymization carries no risk of re-identifying individuals. Data for this research was accessed on July 15th, 2024. The Institutional Review Board (IRB) for Human Research of the University of South Carolina waived the requirements for ethical approval and patient consent in accordance with the Code of Federal Regulations because of the deidentification of data, the lack of an intervention, and the retrospective design. This analysis followed ethical standards in line with the principles of the Helsinki Declaration.

### Mortality-to-incidence ratios

The mortality-to-incidence Ratios (MIR) were calculated as age-adjusted mortality rates divided by age-adjusted incidence rates. To achieve this, incidence rates were calculated as the number of new cases per 100,000 South Carolina population aged 50 and above, first for the 2017–2021 and then for the 2015–2019 periods, to account for possible impacts for the COVID-19 pandemic. Incident cases included new cases of all forms of ADRD over the study period in the registry, and no record of ADRD diagnosis in the preceding 5-year lookback window. Those with ADRD in the years before the study periods were excluded. To identify cases of ADRD and subtypes, the registry uses International Classification of Diseases Codes (ICD-9 and ICD-10). A list of codes for ADRD and subtypes, as captured by the registry are shown in the S1 Table in S1 File. Similarly, mortality rates were calculated as the number of deaths per 100,000 South Carolina population aged 50 and above from 2017 to 2021 and 2015–2019 using multiple-cause coding, where any mention of an ADRD in either the underlying cause of death or as a contributing cause of death was considered an ADRD-related death. The International Classification of Diseases (ICD) codes used to identify cases as shown in the S1 Table in S1 File are the same codes that are applied in determining ADRD-related mortality by the registry. Count data for incidence and mortality are shown in the S2 and S3 Tables in S1 File. Using the direct method of standardization, the age-adjusted incidence and mortality rates were calculated by weighting age-specific incidence and mortality rates to the US 2000 standard population [25]. These age-adjusted estimates were then used in calculating the MIRs.

### Demographic characteristics

Indicators of demographic characteristics (captured within the SC ADRD registry) in this study were sex (male or female), race (White people or Black people), dementia type (Alzheimer’s, vascular, mixed, other), South Carolina health region (Regions 1–8), and rurality (urban or rural). The state health regions are divisions created by the South Carolina Department of Public Health (DPH) to administer environmental and health programs. The SC Regions 1–8 have been described in previous studies [18]. Counties were classified according to the 2023 US Department of Agriculture (USDA) Rural-Urban Continuum Codes. These codes classify the counties into metro or urban counties (codes 1–3) and non-metro or rural (codes 4–9) [26].

### Mapping the mortality-to-incidence ratios

A digitized boundary map for South Carolina showing counties from TIGER/Line shapefiles [27] was used as the base map. This was imported into the Quantum Geographic Information System (QGIS) [28], for mapping MIR at the county level for the state of South Carolina. Mapping of cases by DPH region was done by extracting the DPH regions from the base map. At the regional level, maps by sex and race were also generated.

### Statistical analysis

The age-adjusted MIRs were estimated for the overall population and sub-populations described above. To determine statistically significant differences in the age-adjusted MIRs, 95% confidence intervals (95% Cis) were compared. In a conservative approach, non-overlapping 95% CIs were interpreted to mean that the differences in MIR were statistically significant. The 95% CIs for the MIRs were estimated using the Fay method for directly standardized rates with sparse data [29]. The Fay method uses F-intervals which approximate the exact directly standardized rate intervals. This method is conservative and guarantees nominal coverage (that 95% of intervals constructed using this method will contain the population standardized rate) [29]. MIRs were presented in tables and visualized through mapping with QGIS (version 3.34.10-Prizren) using TIGER/Line shapefiles [27]. All data were deidentified, and all analyses were conducted using SAS version 9.4 (SAS Institute, Cary, NC).

## Results

MIRs by demographic and geographic characteristics, overall and stratified by race are shown in Tables 1 and 2. Table 1 includes the period of the COVID-19 pandemic, while Table 2 excludes this period. For all characteristics, MIRs for Black people were consistently higher than MIRs for White people, irrespective of the period covered. As might be expected, MIRs for periods including the COVID-19 pandemic were consistently higher than MIRs for periods which specifically excluded this time. It is also interesting to note that MIRs higher than 1.00 (in which dementia was only found at death) were only observed among Black people. An example calculation that confirms this interpretation can be found in the S4 Table in S1 File.

**Table 1:** Age-adjusted mortality-to-incidence ratios in South Carolina including COVID19 pandemic time period by demographic and geographic characteristics (2017–2021).

	Age-Adjusted MIR (95% CI)		
	Overall	White people	Black people
**State (SC)**	0.782 (0.781 - 0.784)	0.772 (0.770 - 0.773)	0.819 (0.818 - 0.820)
**Sex**			
Male	0.777 (0.775 - 0.778)	0.771 (0.769 - 0.772)	0.801 (0.800 - 0.802)
Female	0.781 (0.780 - 0.782)	0.768 (0.767 - 0.769)	0.824 (0.822 - 0.825)
**Dementia type**			
Alzheimer’s	0.778 (0.777 - 0.779)	0.774 (0.773 - 0.776)	0.792 (0.791 - 0.793)
Vascular	0.822 (0.817 −0.829)	0.814 (0.807 - 0.820)	0.857 (0.852 - 0.862)
Mixed	0.974 (0.964 - 0.984)	0.924 (0.914 - 0.934)	1.127 (1.117 - 1.137)
Other	0.742 (0.737 - 0.747)	0.685 (0.681 - 0.689)	1.018 (1.012 - 1.025)
**SC Region**			
Region 1	0.856 (0.854 - 0.857)	0.849 (0.847 - 0.850)	0.892 (0.890 - 0.893)
Region 2	0.812 (0.810 - 0.813)	0.814 (0.812 - 0.815)	0.800 (0.799 - 0.801)
Region 3	0.804 (0.802 - 0.805)	0.804 (0.803 - 0.806)	0.807 (0.806 - 0.808)
Region 4	0.806 (0.805 - 0.807)	0.792 (0.791 - 0.794)	0.831 (0.830 - 0.833)
Region 5	0.796 (0.795 - 0.798)	0.758 (0.757 - 0.760)	0.866 (0.864 - 0.867)
Region 6	0.677 (0.676 - 0.679)	0.654 (0.653 - 0.655)	0.816 (0.815 - 0.817)
Region 7	0.770 (0.769 - 0.771)	0.760 (0.758 - 0.761)	0.799 (0.797 - 0.800)
Region 8	0.646 (0.645 - 0.648)	0.616 (0.615 - 0.618)	0.747 (0.745 - 0.748)
**Rurality**			
Rural	0.855 (0.853 - 0.856)	0.838 (0.836 - 0.839)	0.900 (0.888 - 0.891)
Urban	0.767 (0.766 - 0.769)	0.760 (0.759 - 0.761)	0.796 (0.795 - 0.797)

**Table 2:** Age-adjusted mortality-to-incidence ratios in South Carolina excluding COVID19 pandemic time period by demographic and geographic characteristics (2015–2019).

	Age-Adjusted MIR (95% CI)		
	Overall	White people	Black people
**State (SC)**	0.686 (0.685 - 0.687)	0.672 (0.670 - 0.674)	0.741 (0.740 - 0.742)
**Sex**			
Male	0.685 (0.684 - 0.686)	0.676 (0.674 - 0.677)	0.719 (0.718 - 0.721)
Female	0.681 (0.680 - 0.683)	0.664 (0.663 - 0.665)	0.746 (0.745 - 0.747)
**Dementia type**			
Alzheimer’s	0.638 (0.637 - 0.640)	0.630 (0.629 - 0.631)	0.670 (0.668 - 0.671)
Vascular	0.881 (0.875 - 0.888)	0.830 (0.823 - 0.836)	1.022 (1.016 - 1.028)
Mixed	0.859 (0.850 - 0.869)	0.822 (0.812 - 0.831)	0.988 (0.979 - 0.997)
Other	1.113 (1.106 - 1.120)	1.043 (0.986 - 1.083)	1.466 (1.456 - 1.476)
**SC Region**			
Region 1	0.761 (0.760 - 0.763)	0.761 (0.760 - 0.763)	0.758 (0.757 - 0.759)
Region 2	0.715 (0.713 - 0.716)	0.709 (0.708 - 0.711)	0.752 (0.751 - 0.754)
Region 3	0.660 (0.659 - 0.662)	0.651 (0.650 - 0.653)	0.692 (0.691 - 0.693)
Region 4	0.726 (0.725 - 0.727)	0.702 (0.701 - 0.703)	0.771 (0.769 - 0.772)
Region 5	0.680 (0.678 - 0.681)	0.659 (0.658 - 0.661)	0.721 (0.719 - 0.722)
Region 6	0.587 (0.586 - 0.589)	0.563 (0.562 - 0.564)	0.741 (0.740 - 0.742)
Region 7	0.704 (0.702 - 0.705)	0.674 (0.673 - 0.675)	0.802 (0.801 - 0.804)
Region 8	0.578 (0.576 - 0.579)	0.553 (0.552 - 0.554)	0.660 (0.659 - 0.661)
**Rurality**			
Rural	0.735 (0.734 - 0.736)	0.717 (0.715 - 0.718)	0.776 (0.774 - 0.777)
Urban	0.675 (0.674 - 0.676)	0.663 (0.662 - 0.664)	0.728 (0.727 - 0.730)

**Fig 1:**
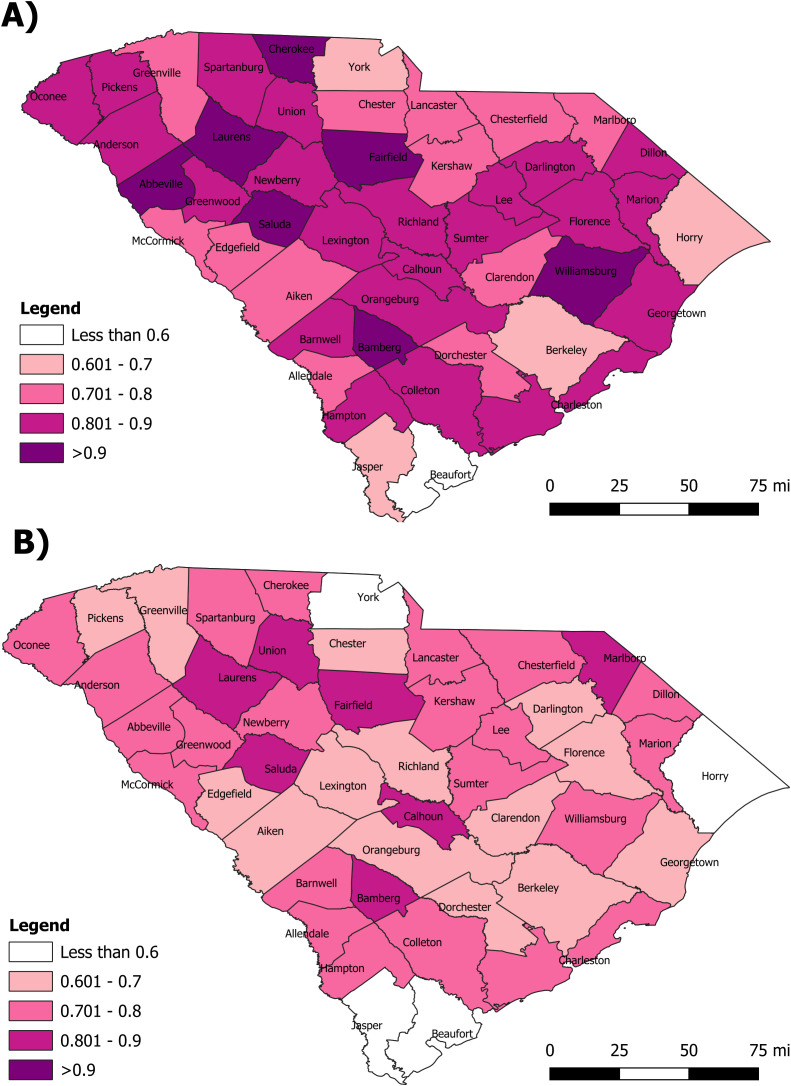
Mortality-to-incidence ratios by county in South Carolina. **A)** MIR maps of South Carolina by county for the period that includes the COVID-19 pandemic (2017 to 2021). **B)** MIR maps of South Carolina by county for the pre-COVID-19 period (2015 to 2019). The lighter the color shade, the lower the MIR quintile and the darker the color shade, the higher the MIR quintile. Source: US Census Bureau TIGER/Line Files publicly available at https://www.census.gov/geographies/mapping-files/time-series/geo/tiger-line-file.html.

Fig 1 provides MIR maps of South Carolina by county. Similar to the trend shown in Table 1 and Table 2, there are a greater number of counties in the highest quintile for the period including the COVID-19 pandemic (Fig 1A). Those counties within the highest MIR quintile include Cherokee, Laurens, Abbeville, Saluda, Fairfield, Bamberg, and Williamsburg (Fig 1A). Only 1 county, Beaufort, was observed in the lowest MIR quintile. In the period excluding the pandemic (Fig 1B), Jasper, Beaufort, and York were observed to have MIRs in the lowest quintile, and there were no counties in the highest quintile. Interestingly, some of the same counties (Laurens, Fairfield, Saluda, and Bamberg) which were in the highest quintile during the pandemic were in the second highest quintile before the pandemic. Union, Calhoun, and Marlboro counties remained in the second highest quintile pre and inclusive of the COVID pandemic.

**Fig 2:**
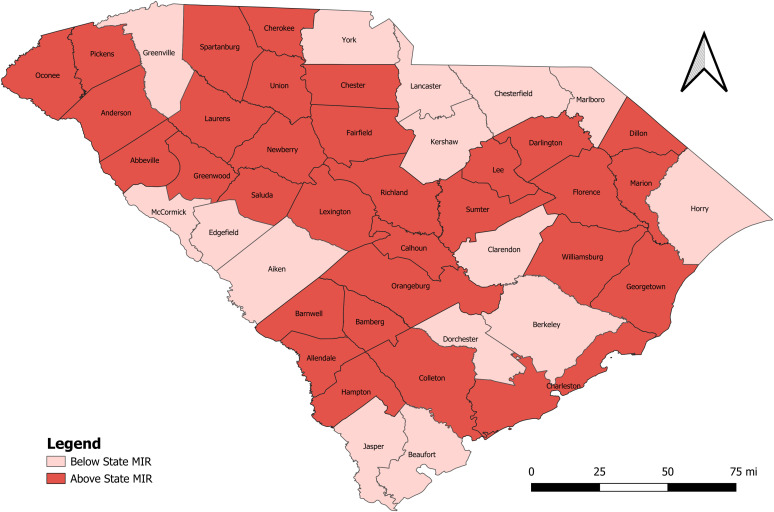
Mortality-to-incidence ratios by county compared to state MIR for 2017 to 2021. The lighter the color shade, the lower the MIR quintile and the darker the color shade, the higher the MIR quintile. Source: US Census Bureau TIGER/Line Files publicly available at https://www.census.gov/geographies/mapping-files/time-series/geo/tiger-line-file.html.

Given the significant impact of the COVID-19 pandemic on MIR estimates, all other figures focused exclusively on the period including this event (2017–2021). Fig 2 highlights those counties with an MIR that exceeded the state MIR. A far greater number of counties were above the state average than were below (31 vs 15).

**Fig 3:**
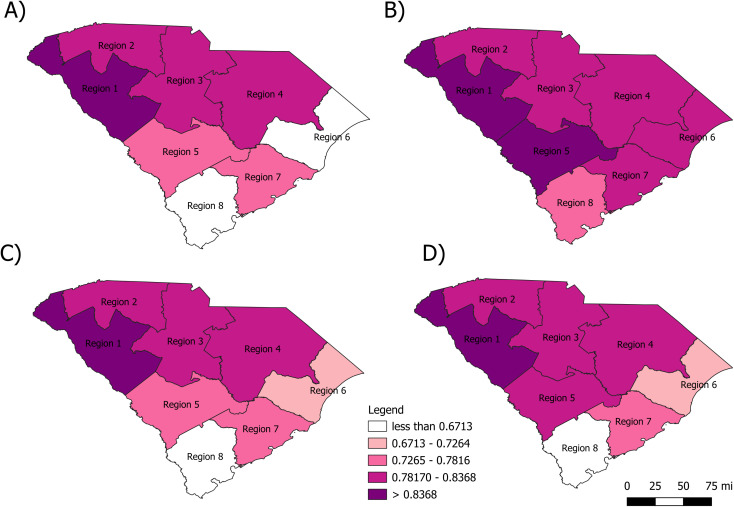
Mortality-to-incidence ratio by region in South Carolina. **A)** MIR by region for White people **B)** MIR by region for Black people **C)** MIR by region for males **D)** MIR by region for females. The lighter the color shade, the lower the MIR quintile and the darker the color shade, the higher the MIR quintile. Source: US Census Bureau TIGER/Line Files publicly available at https://www.census.gov/geographies/mapping-files/time-series/geo/tiger-line-file.html.

Figs 3A and 3B examine MIRs by health regions and race. Regions 1 and 5 fell in the highest quintile of MIRs among Black people while only Region 1 was observed in the highest quintile among White people. There were no regions in the lowest quintile for Black people; however, 2 regions (6 and 8) were observed to fall in this quintile for White people. Similar distribution patterns of MIRs were observed by region for males and females (Figs 3C, 3D). Only region 5 was observed to fall in a higher quintile for females than males.

## Discussion

This study investigated the geospatial distribution of ADRD in South Carolina, focusing on the MIRs across different regions and counties. Using data from the South Carolina Alzheimer’s Disease Registry, ADRD incidence and mortality were examined in relationship with demographic characteristics, including race, region, and county. Overall, Black individuals consistently experienced higher MIRs compared to White individuals, and the COVID-19 pandemic significantly exacerbated this disparity. The regions and counties with the highest MIRs during the pandemic, such as Cherokee and Laurens, were notably more affected.

This unique analysis may be one of the first to apply the MIR for ADRD across South Carolina counties using data from the comprehensive statewide Alzheimer’s Disease Registry. Using the MIR statistic allowed us to examine disease burden by presenting mortality after accounting for incidence [20]. Typical incidence and mortality statistics involve the total population in a particular region, but only individuals diagnosed with a disease may end up dying from the disease. Thus, examining mortality separately may not accurately represent the comprehensive burden of a disease such as ADRD, particularly the burden for racial and ethnic minority populations whose incidence and health experiences may differ from other groups.

MIR results reflect significant inequities at the intersection of race, gender, and geography. Consistent with other research findings, non-Hispanic White adults with ADRD experienced fewer deaths relative to Black people with ADRD within South Carolina [30]. As well, women with ADRD experienced greater mortality than men within the state for both the overall population and for Black residents; Black women with ADRD had the highest recorded number of deaths relative to Black men and non-Hispanic White men and women. Succinctly stated, more Black people, especially women, diagnosed with ADRD within South Carolina died relative to non-Hispanic white people and to the state average. Furthermore, the greatest ADRD related mortality was especially noted among Black people living in rural designated communities. During COVID-19 the number of deaths of people with ADRD was elevated across all populations within South Carolina. Yet, the same pattern of racialized and gendered mortality was consistent: Black people of South Carolina with ADRD experienced greater mortality than non-Hispanic White people and more than the state average.

The disparities in MIR for ADRD observed in this study increased for the period that included the COVID-19 pandemic. This increase may be due to the lack of or reduced access to healthcare and diagnostic services, existing chronic conditions, and the reduced or absence of social support among underserved individuals and populations during the pandemic [31,32]. In addition, regulations and practices such as lockdowns, social distancing and isolation may have contradicted how people with ADRD would typically be managed [33], further worsening the vulnerability of such individuals. In the event of a similar situation, special considerations for managing and caring for individuals with ADRDs could help meet the standard of care they need while protecting them from the dangers of the pandemic.

Our findings of MIR greater than 1 create an opportunity to pause and consider the implications of a ‘diagnosis only after death’ or under-ascertainment of incident cases for Black people of South Carolina. Beyond the need for additional research, this leads to questions about the lack of diagnosis, access to care providers, and access to care services and/or to providers with diagnostic expertise [34,35]. Given the significant mortality differences between Black people, non-Hispanic White people, and state statistics, not only are Black men and women experiencing higher incidence of all types of dementia they are less likely to be diagnosed (and therefore inadequately treated) for vascular and other diagnostic types of dementia. This may also reflect differences in access to the health systems/ providers, providers skilled in diagnosis, lack of insurance, and lack of understanding of the differences between normal aging and ADRD [35–37].

Interventions to address these noted inequities include a range of strategies focused on health professional education and community development. Black Voices in Research has successfully supported health professional education that meaningfully addresses diversity and inclusive excellence for providers and biomedical researchers [38]. Similarly, researchers at Kansas University have created a free online educational program, Aging with Grace, tailored to African-American culture as a way to mitigate the risk of ADRD among Black Americans [39]. Expanding programs such as Dementia Dialogues to the rural population and training healthcare providers to detect ADARD earlier in Black, rural, and marginalized individuals would lead to a reduction in disparities. In addition, improving healthcare access and quality for groups with MIRs such as Black people would be a valuable step towards closing the observed disparities.

Community-based strategies include localized and targeted ADRD programs developed in partnership with community libraries, church communities, with owners of retail establishments such as local barbershops and hair salons, and among established neighborhood associations. The Right to Play organization promotes an internationally successful model of community-based health intervention that leverages an equity-oriented model of health promotion and education among some of the most disenfranchised communities and countries globally. All forms of ‘play’ such as games, sports, poetry, performance, dance, art and music are used to enhance knowledge and skills to improve health among children, their families and their communities [40]. Seamlessly integrated into community and family events, this intervention facilitates community engagement, consistent health communication messaging and a sense of trustworthiness among community members.

Additionally, the World Health Organization (WHO) and the International Association of Age-Friendly Communities provides an integrated Age- and Dementia Friendly Community model intended, in part, to raise awareness, offer education, and share accountability for the health of community members with ADRD [34]. Age- and Dementia Friendly Community interventions include ADRD education focused on people living and working (e.g., grocery store clerks, restaurant wait staff, public transit staff, first responders) in communities to facilitate respectful, equitable, and responsive behaviors to people living with ADRD and their caregivers [34].

In our current study, the MIRs for ADRD were higher than previous estimates of MIR for certain cancers in South Carolina as found by Hébert et al. [18]. These cancers included breast, cervical, colorectal, lung, oral cavity and prostate cancer. Our findings were also higher than estimates of MIR for breast, cervical, colorectal, and prostate cancers in study that explored the relationship between cancer MIR and access to federally qualified health centers in South Carolina [20]. While our study found higher MIRs for ADRD, it is important to note that comparing MIR across different disease conditions should be done with caution. Differences in MIR are not solely due to lethality but also reflect differences in diagnosis or case-ascertainment, death certificate practices, and survival duration after diagnosis. The higher MIR for ADRD as found in this study could be a combination of case under-ascertainment, differences in death certification, and lethality.

There are some limitations which should be considered in the interpretation of our findings. Most notably, this work represents an ecological analysis based upon population statistics. While we can hypothesize potential factors which may influence our observations, individual-level information on specific risk factors for diagnosis and survival are needed to ascertain a true association. In addition, we are unable to draw causal conclusions because of the cross-sectional nature of the analysis, and the contrast of 2017–2021 with 2015–2019 could blur a clean pre and post pandemic interpretation. Nevertheless, our findings provide an excellent indicator for sub-groups and populations who are experiencing a higher burden of ADRD disease. Another limitation in this study is the possibility of under-diagnosis or under-ascertainment of incident cases in some populations, which could partly influence estimated MIRs. Nonetheless, the SCADR is one of the four statewide ADRD registries in the U.S. and the most comprehensive in terms of data. There are also limits to the generalizability of these findings. Findings of this study are generalizable to regions with similar demographics and healthcare systems, therefore care should be taken when applying them to regions with different characteristics. The study’s findings emphasize the importance of comprehensive public health registries to assess and address disparities in ADRD. County-level MIRs provide a nuanced view of ADRD burden, demonstrating that mortality rates, especially among Black populations, are disproportionately higher in certain regions. The results suggest a need for increased awareness, targeted community education, and advocacy to ensure better care for vulnerable populations affected by ADRD in South Carolina. This study also underscores the vital role of the initiatives that target rural and underserved regions, e.g., the University of South Carolina Brain Health Network. These initiatives can address disparities in ADRD care, particularly in rural regions more strongly affected by the disease. Access to diagnosis and treatment in underserved areas is crucial for reducing the higher MIRs observed among the most vulnerable populations. Finally, registries such as the South Carolina Alzheimer’s Disease Registry have tremendous potential to impact public health surveillance and applied research, and inform educational programming, interventions, and policy. Examining MIRs of ADRD in relation to COVID-19 revealed striking differences by county and particularly regarding racial and gender differences. These important findings can inform future educational interventions with community members, caregivers, and healthcare providers. In addition, using this data to promote awareness of ADRD in high-risk counties of the state with policymakers, legislators, and community leaders can help with advocacy for improved care and funding. Future studies should apply the MIR to evaluate ADRD related policies and identify regions that will need a policy or resource boost to improve the ascertainment and management of ADRD. In addition, future studies could also combine survival analysis to verify whether MIR differences stem from prognostic differences, further disentangling the contributions of detection and survival bias. Further research on how interventions would directly impact disparities in ADRD will also be a valuable contribution to developing targeted interventions.

## Supporting information

S1 File Contains the supporting tables (S1 – S4 Tables). (DOCX)
