# Supplementary material for: Mapping the mortality-to-incidence ratios of Alzheimer’s Disease and Related Dementias (ADRDs): Evidence from the South Carolina Alzheimer’s disease registry
Source: PLoS One. 2025 Dec 29;20(12):e0339785. doi: 10.1371/journal.pone.0339785 (PMC12747324; doi:10.1371/journal.pone.0339785)
Supplement: S1 File — (DOCX) [file pone.0339785.s001.docx]

S1Table. List of International Classification of Diseases (ICD) Codes as captured by the South Carolina Alzheimer’s Disease Registry (SCADR)

| Dementia Type | ICD-9 codes | ICD-10 codes |
| --- | --- | --- |
| Alzheimer’s Disease | 290.0-290.3  290.8-290.9  331.0 | F03.90 – F03.91  G30.0 – G30.9 |
| Vascular Dementia | 290.4-290.43  435-438 | FO1.50 – F01.51  G45.0 – G45.9  I67.0-I67.9  I69.00-I69.998 |
| Mixed Dementia | Both Alzheimer’s Disease and Vascular Dementia | Both Alzheimer’s Disease and Vascular Dementia |
| Frontotemporal Dementia | 331.19 | G31.09 |
| Dementia with Lewy bodies | 331.82 | G31.83 |
| Picks disease | 331.11 | G31.01 |
| Dementia with other conditions | 294.1 | F02.80-F02.81 |
| Alcohol induced Dementia | 291.2 | F10.27 – F10.97 |
| Drug induced dementia | 292.82 | Drug-induced dementia |

S2 Table. Counts of new cases and mortality in the SCADR from 2017 - 2021

|  | Overall | | White People | | Black People | |
| --- | --- | --- | --- | --- | --- | --- |
|  | Incident | Death | Incident | Death | Incident | Death |
| **State (SC)** | 62,590 | 47,609 | 47,299 | 35,575 | 15,291 | 12,034 |
| **Sex** |  |  |  |  |  |  |
| Male | 25,846 | 19,335 | 19,428 | 14,443 | 6,418 | 4,892 |
| Female | 36,743 | 28,271 | 27,870 | 21,131 | 8,873 | 7,140 |
| **Dementia type** |  |  |  |  |  |  |
| Alzheimer’s | 52,112 | 39,347 | 39,452 | 29,743 | 12,660 | 9,604 |
| Vascular | 3,496 | 2,766 | 2,307 | 1,812 | 1,189 | 954 |
| Mixed | 1,669 | 1,585 | 1,200 | 1,084 | 469 | 501 |
| Other | 5,313 | 3,911 | 4,340 | 2,936 | 973 | 975 |
| **SC Region** |  |  |  |  |  |  |
| Region 1 | 6,864 | 5,752 | 5,599 | 4,656 | 1,265 | 1,091 |
| Region 2 | 12,873 | 10,222 | 10,839 | 8,655 | 2,034 | 1,567 |
| Region 3 | 11,573 | 9,021 | 8,470 | 6,654 | 3,103 | 2,367 |
| Region 4 | 8,464 | 6,656 | 5,152 | 4,013 | 3,312 | 2,643 |
| Region 5 | 3,762 | 2,929 | 2,380 | 1,772 | 1,382 | 1,157 |
| Region 6 | 6,753 | 4,351 | 5,775 | 3,585 | 978 | 766 |
| Region 7 | 8,127 | 6,043 | 5,887 | 4,316 | 2,240 | 1,727 |
| Region 8 | 4,174 | 2,635 | 3,197 | 1,924 | 977 | 711 |
| **Rurality** |  |  |  |  |  |  |
| Rural | 10,974 | 9,161 | 7,203 | 5,908 | 3,771 | 3,253 |
| Urban | 51,616 | 38,448 | 40,096 | 29,667 | 11,520 | 8,781 |

S3 Table. Counts of new cases and mortality in the SCADR from 2015 - 2019

|  | Overall | | White | | Black | |
| --- | --- | --- | --- | --- | --- | --- |
|  | Incident | Death | Incident | Death | Incident | Death |
| **State (SC)** | 64,064 | 43,429 | 50,092 | 33,295 | 13,972 | 10,134 |
| **Sex** |  |  |  |  |  |  |
| Male | 25,619 | 17,157 | 19,832 | 13,092 | 5,787 | 4,065 |
| Female | 38,443 | 26,266 | 30,259 | 20,198 | 8,184 | 6,068 |
| **Dementia type** |  |  |  |  |  |  |
| Alzheimer’s | 54,746 | 34,423 | 43,004 | 26,774 | 11,742 | 7,649 |
| Vascular | 3,509 | 3,022 | 2,466 | 2,012 | 1,043 | 1,010 |
| Mixed | 1,630 | 1,375 | 1,203 | 975 | 427 | 400 |
| Other | 4,179 | 4,609 | 3,419 | 3,534 | 760 | 1,075 |
| **SC Region** |  |  |  |  |  |  |
| Region 1 | 7,287 | 5,515 | 6,112 | 4,630 | 1,175 | 885 |
| Region 2 | 13,394 | 9,494 | 11,710 | 8,269 | 1,684 | 1,225 |
| Region 3 | 12,327 | 8,036 | 9,412 | 6,076 | 2,915 | 1,960 |
| Region 4 | 8,751 | 6,283 | 5,503 | 3,843 | 3,248 | 2,440 |
| Region 5 | 3,859 | 2,617 | 2,545 | 1,683 | 1,314 | 934 |
| Region 6 | 6,593 | 3,764 | 5,676 | 3,101 | 917 | 663 |
| Region 7 | 7,834 | 5,431 | 6,035 | 4,005 | 1,799 | 1,426 |
| Region 8 | 4,019 | 2,289 | 3,099 | 1,688 | 920 | 601 |
| **Rurality** |  |  |  |  |  |  |
| Rural | 11,639 | 8,485 | 7,980 | 5,683 | 3,659 | 2,802 |
| Urban | 52,425 | 34,944 | 42,112 | 27,612 | 10,313 | 7,332 |

Sample Calculation to Illustrate an MIR of greater than 1

Using Mixed Dementia for Black people as an example, the table below shows a sample calculation to illustrate MIR greater 1 (S1 Table). The MIR is estimated by dividing mortality by incidence, in this case, the age-adjusted mortality by the age-adjusted incidence. As shown in the table below, because mortality exceeds incident cases, the ratio of mortality to incidence will exceed 1. Plausible interpretations will include under-ascertainment of cases or a higher mortality than diagnosis.

S4 Table. Sample Calculation to Illustrate MIR > 1

| Incident cases | Deaths | Age-adjusted incidence per 100,0000 | Age-adjusted mortality per 100,0000 | MIR | Interpretation |
| --- | --- | --- | --- | --- | --- |
| 469 | 501 | 127 | 143 | 1.13 | An MIR of greater than is reflective of more deaths than cases. As shown, the mortality rate exceeds incidence rates. Plausible explanations include under-ascertainment of incident cases or a record of diagnosis only at death |
